# Inactivation and Damage of Histamine-Forming Bacteria by Treatment with High Hydrostatic Pressure

**DOI:** 10.3390/foods9030266

**Published:** 2020-03-02

**Authors:** Yi-Chen Lee, Yung-Hsiang Tsai, Shao-Lan Chen, Hsien-Feng Kung, Osamu Arakawa, Cheng-I Wei

**Affiliations:** 1 Department of Seafood Science, National Kaohsiung University of Science and Technology, Kaohsiung 811, Taiwan; yhtsai01@seed.net.tw (Y.-H.T.); ap92425@gmail.com (S.-L.C.); 2 Department of Pharmacy, Tajen University, 907 Pingtung, Taiwan; khfeng@mail.tajen.edu.tw; 3 Graduate School of Fisheries and Environmental Sciences, Nagasaki University, 852-8521 Nagasaki, Japan; arakawa@nagasaki-u.ac.jp; 4 Department of Nutrition and Food Science, University of Maryland, College Park, MD 20742, USA; wei@umd.edu

**Keywords:** high hydrostatic pressure, histamine-forming bacteria, inactivation, SEM

## Abstract

The inactivation and damage of histamine-forming bacteria (HFB), *Enterobacter aerogenes* and *Staphylococcus capitis*, in a 0.1 M potassium phosphate buffer (pH 6.8) and marlin meat slurry by high hydrostatic pressure (HHP) treatments were studied using viability measurement and scanning electron microscopy (SEM). HHP treatments showed first order destruction kinetics to *E. aerogenes* and *S. capitis* during the pressure holding period. HFB in marlin meat slurry had higher D values and were more resistant to HHP treatments than in phosphate buffer. In phosphate buffer, *E. aerogenes* had higher D values than *S. capitis* at >380 MPa of pressure, whereas the reverse trend was noticed at lower pressures (<380 MPa). In marlin meat slurry, *S. capitis* had a higher D value than *E. aerogenes* at the same treatment pressure, indicating that *S. capitis* was more resistant to HHP treatment. To our knowledge, this is the first report to demonstrate that HHP can be used to inactivate HFB, *E. aerogenes,* and *S. capitis*, by causing disruption to bacterial cell membrane and cell wall as demonstrated by SEM micrographs.

## 1. Introduction

Histamine fish poisoning, or scombroid poisoning, is an allergy-like form of food poisoning resulting from the consumption of mishandled scombroid fish that contains high contents of histamine [1]. Histamine is generated mainly by the decarboxylation of histidine in fish muscle through the actions of histidine decarboxylases of histamine-forming bacteria (HFB) that are present in the seafood. HFBs have been isolated from scombroid fish and other seafood products, as well as fermented foods such as wine, sausage, and cheese [2]. Two different types of histidine decarboxylase have been distinguished in these bacteria: Pyruvoyl-dependent enzymes and pyridoxal-5’-phosphate-dependent enzymes [3]. The pyruvoyl-dependent enzymes are encountered in Gram-positive bacteria such as lactic acid bacteria and *Staphylococcus* spp., producing histamine in fermented products including wine, cheese, douchi, mustard pickle, and fish sauce [4,5]. On the contrary, the pyridoxal-5’-phosphate-dependent enzymes are identified in Gram-negative enteric bacteria such as *Enterobacter aerogenes*, *Raoultella orithinolytica*, and *Morganella morganii*, producing histamine in the fish [5,6].

High hydrostatic pressure (HHP) is a nonthermal technology for food pasteurization and preservation [7]. In a commercial setting, HHP was used at a pressure above 300 MPa to kill spoilage and pathogenic microorganisms for shelf-life extension and safety improvement of jams, fruit juices, guacamole, meats, dairy and egg products, and seafood [8,9]. The usage of HHP treatment to preserve the freshness of food was also shown to not affect some of the food quality characteristics such as the color, natural flavor, and nutrients [9,10]. However, HHP technology can have some disadvantages on the quality of fish including color changes with higher L* values associated with higher opacity, harder textures, and lipid oxidation [11,12]. HHP treatment was reported to be capable of killing *Listeria monocytogenes*, *Escherichia coli*, and *Vibrio parahaemolyticus* through morphological damages to both the internal and external structures [13,14]. A treatment at a pressure of >300 MPa can cause irreversible denaturation of enzymes and proteins to affect the integrity of the cell membrane, lowering protein biosynthesis and inhibit protein repairs, and ultimately resulting in cell membrane rupture, excretion of internal substances, and bacterial death [14,15].

HHP treatments can successfully control bacteria, but there are few data of inactivation kinetics on bacteria, especially concerning bacterial species and medium effects. In food processing, D (decimal reduction time) -value is the most frequently used kinetic parameter. Since only limited information was available on the inactivation effect and morphological damage of HFBs by HHP treatment, the aim of this study was to find out the inactivation kinetics of HHP processing on HFB, *S. capitis,* and *E. aerogenes*, in a 0.1 M phosphate buffer (pH 6.8) and marlin meat slurry, and to evaluate whether morphological damages occurred in HHP-treated HFB cells.

## 2. Materials and Methods

### 2.1. Bacterial Culture Preparation

Stock cultures of *E. aerogenes* and *S. capitis* were isolated from marlin meat [16] and dried milkfish [17], respectively. Briefly, both histamine-forming isolates were picked from differential agar plates (histamine-forming bacterium isolation agar) and identified by amplifying and sequencing approximately 1400 bp of the 16S ribosomal DNA (rDNA) [16,17]. The cultures were maintained in our laboratory on Trypticase Soy Agar (Difco Becton-Dickinson Co., Sparks, MD, USA) at 4 °C.

### 2.2. Preparation of E. aerogenes and S. capitis in Phosphate Buffer and Marlin Meat Slurry

One loopful of *E. aerogenes* or *S. capitis* was inoculated into the Trypticase Soy Broth (TSB) tube (5 mL) and incubated at 35 °C for 12 h; then 100 μL aliquot of the bacterial culture was added to 100 mL sterile TSB medium at 35 °C for 24 h. The cultured broth was centrifuged at 8000× *g* for 15 min at 4 °C and the bacterial pellet was washed and resuspended in 0.05 M potassium dihydrogen phosphate buffer (pH 7.0). The bacterial suspension was then adjusted to a concentration of 10 ^9^ CFU/mL.

Fresh marlin flesh was purchased from a local market in Kaohsiung City, Taiwan and transported in ice to the laboratory immediately. After washing with a 75% ethanol solution for 1 min and rinsing with sterile water, the flesh was ground to mince in a sterile food homogenizer. The fish mince was then blended with 0.1% peptone water (1:4; w/w) for 2 min in a blender (Omni International, Waterbury, CT, USA). Both the sterile potassium phosphate buffer (0.1 M, pH 6.8, 99 mL) and the marlin meat slurry (99 mL) were inoculated with 1 mL of HFB inoculum (10^9^ CFU/mL) to get at 10^7^ CFU/mL as the final concentration. The tested samples were added to sterile vacuum bags in 10-mL portions, vacuum packaged and heat-sealed, and then subject to HHP treatments immediately.

### 2.3. High Hydrostatic Pressure Treatment

Test bags in triplicate were treated with a laboratory model of high pressure processing system (BaoTou KeFa, High Pressure Technology, Co. Ltd., Baotou, China) at 200 to 500 MPa for 0 to 10 min at room temperature (25 °C). This high pressure processing system having a 6.2-L chamber can be operating at up to 600 MPa at a pressure increase rate of approximately 300 MPa/min and the release times of less than 20 s at all pressures. Water was used as a pressure transmission medium in this study, and the reported pressurization times did not include the time for pressure increase and release. An untreated bag placed in ice water at ambient pressure (0.1 MPa) served as a control. Samples subject to pressure treatment were stored in ice water and immediately used for bacterial counting and SEM analyses.

### 2.4. Enumeration of HFB Surviving Cells and Decimal Reduction Time

Both the HHP treated and nontreated bacterial suspensions in phosphate buffer or fish slurry were 10-fold serially diluted in a sterile phosphate buffer (0.05 M, pH 7.0). Aliquots (1.0 mL) of the diluents were mixed in petri dishes with 15 mL TSA (Difco) at 45–50 °C. After the agar medium was solidified in a laminar flow hood, the plates were transferred to an incubator and incubated at 30 °C for 24–48 h. Bacterial colonies numbers on the plates were counted. Data from triplicate samples were presented as mean ± standard deviation.

The linear first order reaction was used as follows to determine the pressure destruction kinetics of HFB during the pressure-hold time phase with log numbers of survivors:

Log (*N/N_0_*) = −1/D × t

where *N_0_* is the initial number of HFB in untreated samples, *N* is the surviving number of HFB after pressure treatment for time t (min). The D-value or decimal reduction time is the treatment time at any given pressure causing 90% reduction of the HFB population, i.e., resulting in one logarithm reduction of the microbial population. D-value was obtained by the negative reciprocal slope of the log (*N*/*N*_0_) vs. time.

The decimal logarithm of D-values vs. pressure was plotted to determine the pressure sensitivity of the D-values.

### 2.5. Scanning Electron Microscopy (SEM) Analysis

HFB cells in a 0.1 M phosphate buffer (pH 6.8) were harvested from pressure-treated (500 MPa for 10 min) and nontreated suspensions via centrifugation at 5000 rpm for 20 min. After two washes with phosphate buffer, the pellets were resuspended in 1 mL of phosphate buffer and then filtered through Millipore membranes (0.22 μm MF—Millipore, GSWP; Millipore Corp., Billerica, MA, USA). Cells on the filters were fixed with 10 mL of 1.5% glutaraldehyde/0.1 M phosphate buffer (pH 6.8) and left overnight for drying at 4 °C. After the cells on the membranes were washed three times with phosphate buffer for 10 min, they were post-fixed for 90 min in 1% osmium tetroxide (OsO_4_) and then rinsed with phosphate buffer twice (10 min per rinse). The cells on the membranes were then dehydrated in a series of 10 mL ethanol solutions (35%, 50%, 60%, 70%, 85%, 90%, 95%, 100%, and 100% ethanol, 15 min each), immersed in isopentyl acetate and finally in carbon dioxide medium for critical point drying using a critical point dryer (HCP-2, Hitachi Koki Co., Ltd., Ibaragi, Japan). The dried membranes were then mounted on scanning electron microscope stubs, sputter-coated with a thin film of gold-palladium, and then observed by the SEM (S4700, Hitachi Koki Co., Ltd., Ibaragi, Japan) operating at 15 kV voltage. SEM photomicrographs were taken from different regions of the same dried specimen.

## 3. Results and Discussion

### 3.1. Inactivation Kinetics of HHP Treatment on Histamine-Forming Bacteria in Phosphate Buffer

**Figure 1:**
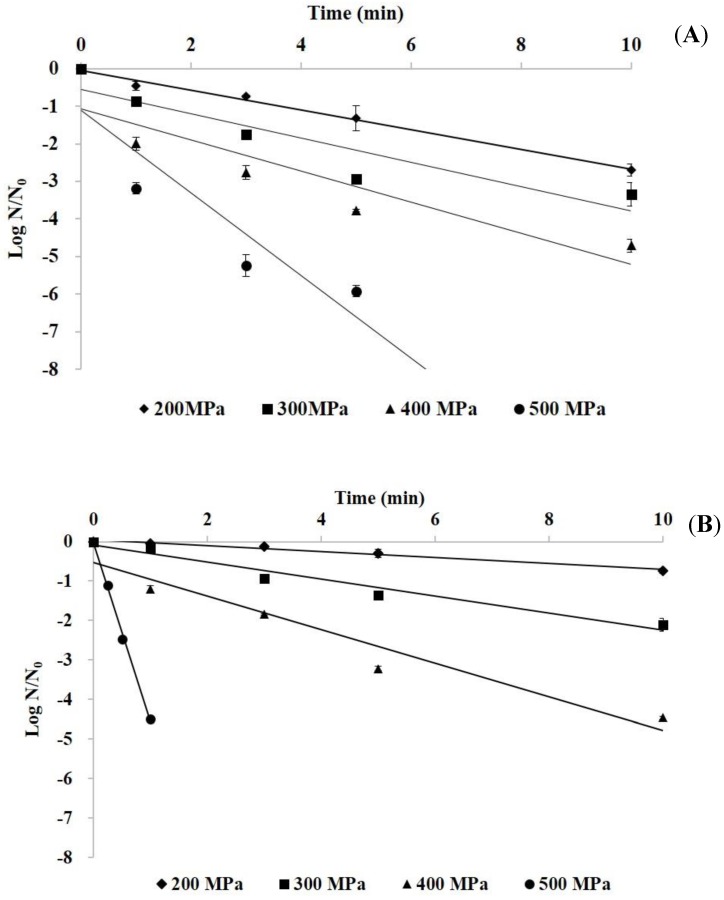
The survival curves of *E. aerogenes* (**A**) and *S. capitis* (**B**) in phosphate buffer by high hydrostatic pressure (HHP) treatment at 200–500 MPa for up to 10 min.

Figure 1 shows the survival curves of *E. aerogenes* and *S. capitis* in phosphate buffer following HHP treatment at 200–500 MPa for up to 10 min. Both the treatment pressure and the holding time influenced the destruction of the bacteria. The sharper survival curves at higher pressures than at lower pressures indicated that the destruction rate was greater at higher pressures. The first order model fits the destruction kinetics of HHP treatment on HFB during the hold period, indicating that pressure destruction of *E. aerogenes* and *S. capitis* complied with the semi-logarithmic model. From the survival curves, the D-values could be calculated and used for comparison of microbial resistance to HHP treatment or the effectiveness of such treatment on microbial destruction. The computed D-values and R squared parameters of *E. aerogenes* and *S. capitis* in phosphate buffer showed that *S. capitis* had higher D-values (13.4 and 4.65 min, respectively), and therefore more resistant, than *E. aerogenes* (3.82 and 3.08 min, respectively) when treated with HHP at 200 and 300 MPa (Table 1). However, as the pressure level was elevated to 400 MPa, the difference in the D-values diminished, with *E. aerogenes* having a value of 2.42 min and *S. capitis* of 2.35 min. At 500 MPa, the D-values were 0.92 and 0.22 min for *E. aerogenes* and *S. capitis*, respectively.

**Figure 2:**
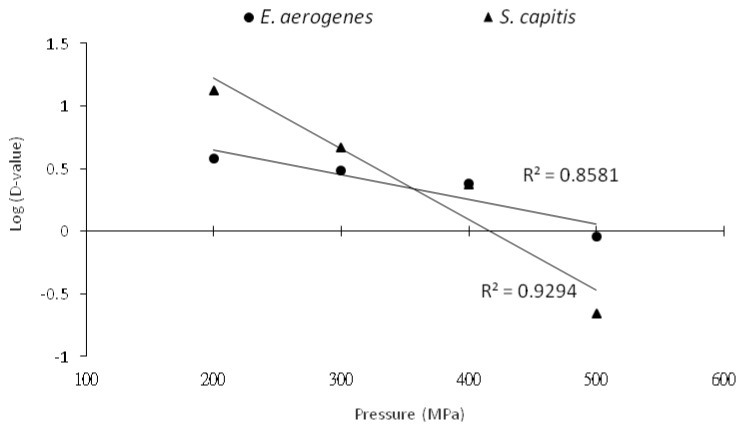
HHP decimal reduction time curves for *E. aerogenes* and *S. capitis* in phosphate buffer.

The HHP decimal reduction time curves as obtained by charting the decimal logarithm of D-values vs. pressure showed a crossover pressure point of 380 MPa for the two bacterial species, indicating that *E. aerogenes* in phosphate buffer was more resistant than *S. capitis* to HHP destruction at pressures >380 MPa (Figure 2). This also means that it would require shorter holding times to destroy *E. aerogenes* than *S. capitis* at lower treatment pressures of <380 MPa.

The inactivation of *Escherichia coli* in saline solution by HHP treatments was followed by first order kinetics, with D-values of 25.9 min at 200 MPa, 8.0 min at 250 MPa, 2.5 min at 300 MPa, and 0.8 min at 350 MPa [18]. Patterson et al. [19] demonstrated that *E. coli* in phosphate buffer had a D-value of 13 min at 700 MPa; and Furukawa et al. [20] showed a calculated D-value of 143 min at 100 MPa for *E. coli* in tryptic soy broth. Both the test medium and the bacterial strains used for HHP treatment contributed to differences in the D-values of treated *E. coli* or *Listeria monocytogenes* [21]. Tassou et al. [22] reported the inactivation of *S. aureus* in phosphate buffer, with D-values of 21.1, 17.3, 7.0, 1.6, and 0.6 min for HHP treatments at 300, 350, 400, 450, and 500 MPa, respectively. However, the D-value of *S. aureus* at 350 MPa was about 10 min in phosphate buffer [23].

### 3.2. Inactivation Kinetics of HHP Treatment on Histamine-Forming Bacteria in Marlin Meat Slurry

**Figure 3:**
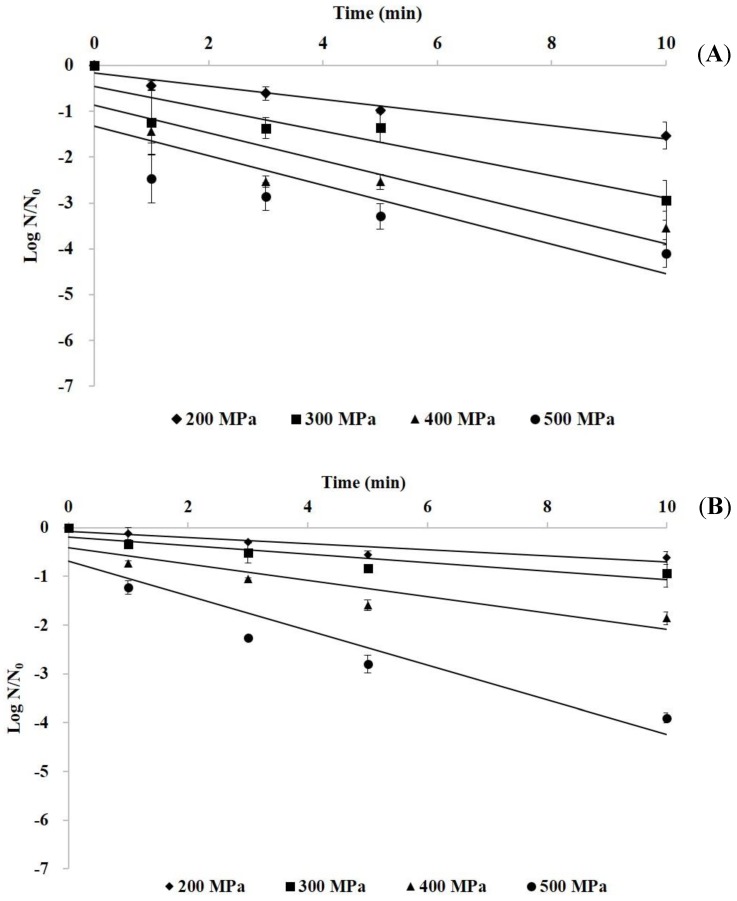
The survival curves of *E. aerogenes* (**A**) and *S. capitis* (**B**) in marlin meat slurry by HHP treatment at 200–500 MPa for up to 10 min.

The inactivation kinetics during HHP treatment (200–500 MPa for 0–10 min) of *E. aerogenes* and *S. capitis* in marlin meat slurry are shown in Figure 3. The logarithm of the surviving *E. aerogenes* and *S. capitis* in the meat slurry linearly decreased with the increase of pressure time, indicating that the HHP inactivation followed adequately first order kinetics. The computed D-values of HHP-treated bacteria in meat slurry showed a higher D-value at lower treatment pressure for both bacterial species, and the treated *S. capitis* at 200, 300, and 400 MPa had a higher D-value than *E. aerogenes* (Table 1). However, at the treatment pressure of 500 MPa, the treated *E. aerogenes* had a D-value of 2.99 min and the 2.82 min for *S. capitis*.

**Figure 4:**
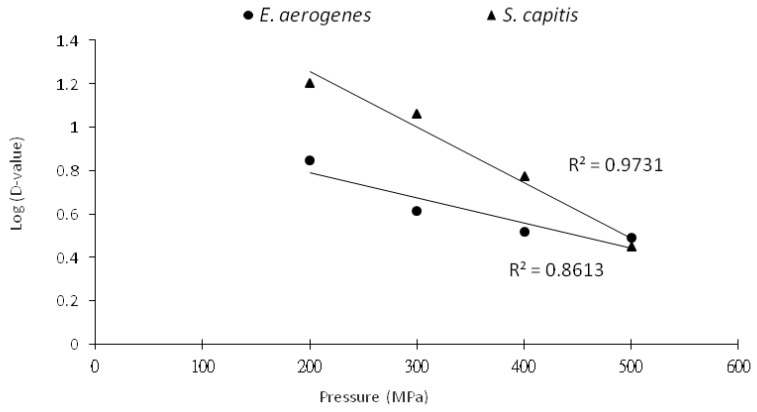
HHP decimal reduction time curves for *E. aerogenes* and *S. capitis* in marlin meat slurry.

The HHP decimal reduction time curves for both bacterial species showed a crossover pressure point at 500 MPa (Figure 4). Thus, *S. capitis*, in general, had a higher D-value than *E. aerogenes* in marlin meat slurry because it was more resistant to pressure treatment than *E. aerogenes*. Food processing using a pressure treatment at <500 MPa would take a longer holding time to carry out the same damaging effect to the contaminated *S. capitis* as to *E. aerogenes*.

Hoover et al. [24] reported that Gram-positive bacteria are in general regarded to be more resistant than Gram-negative bacteria to HHP treatment. In addition, the small and spherical bacteria were generally regarded as more tolerant to HHP treatment than large and rod-shaped bacteria [15]. Therefore, Gram-positive and cocci bacteria are more resistant to HHP treatment than Gram-negative and rod-shaped bacteria. The results shown in this study exhibited a similar finding that the G(+) *S. capitis* in marlin meat slurry was more resistant to high pressure treatment than the G(−) *E. aerogenes*.

In this study, both the *S. capitis* and *E. aerogenes* in marlin meat slurry had higher D-values than those in phosphate buffer for all the pressure treatment conditions (Table 1), indicating that the HFB were more resistant to pressure treatment in marlin meat slurry than in phosphate buffer. Many intrinsic and environmental parameters, especially the nature of the suspension medium, influence the resistance of microorganisms to pressure treatment. Simpson and Gilmour [25] reported that bacteria existing in nutrient-rich media had great survival ability to high pressure treatment because the media contained nutrients that are essential for repairing or substances that may provide protection against damage. Microorganisms in food systems were more resistant to HHP treatment than in buffer solution, while such resistance ability to pressure treatment increased as the water activity decreased [26]. Fish matrix was reported to have higher protective effect to spoilage bacteria than the phosphate buffer at pressures below 550 MPa [27]. Patterson [28] also stated that some food constituents such as lipids, proteins, carbohydrates, and salt can have a protective effect for the microbial cells. Therefore, the HPB cells in marlin meat slurry are more protected against HHP treatment due to protein and lipid contents.

### 3.3. SEM Micrographs of Histamine-Forming Bacteria after Exposure to HHP Treatment

**Figure 5:**
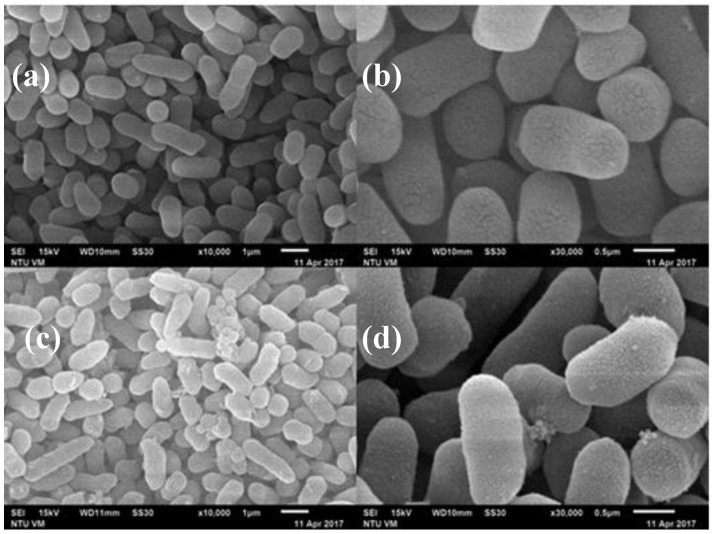
SEM micrographs of *Enterobacter aerogenes* cells in phosphate buffer. (**a**) and (**b**) Untreated cells; (**c**) and (**d**), cells treated with 500 MPa for 10 min.

**Figure 6:**
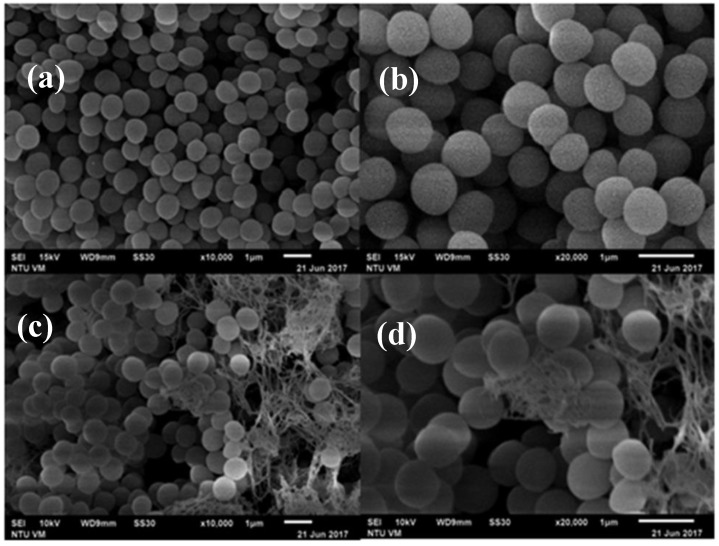
SEM micrographs of *Staphylococcus capitis* cells in phosphate buffer. (**a**) and (**b**) Untreated cells; (**c**) and (**d**), cells treated with 500 MPa for 10 min.

Figure 5 and Figure 6 are the SEM micrographs of *E. aerogenes* and *S. capitis* in phosphate buffer following HHP treatment at 500 MPa for 10 min, respectively. Compared to the untreated cells (Figure 5a,b and Figure 6a,b), damages of cellular envelopes and intracellular structures occurred with *E. aerogenes* and *S. capitis* after HHP treatment (Figure 5c,d and Figure 6c,d, respectively). At lower magnification (Figure 5c and Figure 6c), the treated bacteria showed some roughness features on the cell wall, the occurrence of pimples-like damages, and swellings that resulted in some cells being compressed and other shattered. Similar findings were also reported previously with the treated *Listeria* cells at 400 MPa for 10 min and *V. parahaemolyticus* at 300 MPa for 10 min [14,29]. Closer observations of the treated bacterial cells in Figure 5d and Figure 6d showed the presence of broken cell walls and perforation, and the loss of plasma membrane and cytoplasm content.

Ritz et al. [30] employed SEM to show the presence of bud scars on cellular surface after pressure treatment of *L. monocytogenes*, and the loss of membrane integrity in most of the bacterial cells. Mackey et al. [31], by using electron micrographs, showed that bacterial cells of different genera had different resistance to high pressure treatment, and pressure treatment led to changes in cellular morphology and intracellular enzyme activity. SEM examination of pressure-treated *S. aureus* and *E. coli* O157:H7 at 200 and 400 MPa showed increases in volume and view area of the microorganisms [29], possibly attributed to phase transition of phospholipid bilayer and denaturation of bound proteins on the cell membrane due to HHP treatment. Marx et al. [32] showed perforation damages on the cell membrane and cell wall, and scars on cells surface of *Saccharmyces cerevisiae* in apple juice after HHP treatment at 600 MPa for 7 min. Recently, Wang et al. [14] indicated that the cause of damage to bacterial membrane by HHP treatment is one of the most important underlying mechanisms of HHP inactivation of bacterial pathogens. All these studies supported the findings that HHP treatment of bacterial cells causes damages to cell membrane permeability, loss of membrane integrity, cellular swelling, and eventually cell death. The results of this study suggest that the damage site of *E. aerogenes* and *S. capitis* by HHP treatment could be the cell membrane or cell wall.

## 4. Conclusions

This study, with the objective of investigating the inactivation of HFB using HHP treatment, showed that HHP can be applied to inactivate histamine-forming bacteria *S. capitis* and *E. aerogenes* by damaging the cell wall and cell membrane. The results showed that HFB in marlin meat slurry were more resistant to HHP treatment than in phosphate buffer. In marlin meat slurry, *S. capitis* was more resistant to HHP treatment than *E. aerogenes.*

## Figures and Tables

**Table 1:** The inactivation kinetics of high hydrostatic pressure on *E. aerogenes* and *S. capitis* in phosphate buffer and marlin meat slurry.

Pressure (MPa)	Slope	D-Value (min) ^a^	R^2^
In phosphate buffer			
*E. aerogenes*			
200	−0.26	3.82	0.98
300	−0.33	3.08	0.86
400	−0.41	2.42	0.83
500	−1.10	0.92	0.85
*S. capitis*			
200	−0.07	13.4	0.98
300	−0.22	4.65	0.96
400	−0.43	2.35	0.94
500	−4.52	0.22	0.99
In marlin meat slurry			
*E. aerogenes*			
200	−0.14	7.00	0.95
300	−0.24	4.11	0.86
400	−0.31	3.26	0.85
500	−0.33	2.99	0.83
*S. capitis*			
200	−0.06	16.0	0.86
300	−0.09	10.9	0.85
400	−0.17	5.87	0.85
500	−0.36	2.82	0.89

^a^ D: Decimal reduction time (min); R^2^: Regression coefficient.
